# Future of work in 2050: thinking beyond the COVID-19 pandemic

**DOI:** 10.1186/s40309-022-00210-w

**Published:** 2022-12-10

**Authors:** Carlos Eduardo Barbosa, Yuri Oliveira de Lima, Luis Felipe Coimbra Costa, Herbert Salazar dos Santos, Alan Lyra, Matheus Argôlo, Jonathan Augusto da Silva, Jano Moreira de Souza

**Affiliations:** 1 Program of Systems and Computer Engineering, Universidade Federal do Rio de Janeiro, Rio de Janeiro, Brazil; 2 Centro de Análises de Sistemas Navais (CASNAV), Rio de Janeiro, Brazil

**Keywords:** Work, Employment, Social welfare, Automation, Future, Foresight, Technological change, Technological unemployment, Scenario, COVID-19

## Abstract

Work has been continuously changing throughout history. The most severe changes to work occurred because of the industrial revolutions, and we are living in one of these moments. To allow us to address these changes as early as possible, mitigating important problems before they occur, we need to explore the future of work. As such, our purpose in this paper is to discuss the main global trends and provide a likely scenario for work in 2050 that takes into consideration the recent changes caused by the COVID-19 pandemic. The study was performed by thirteen researchers with different backgrounds divided into five topics that were analyzed individually using four future studies methods: Bibliometrics, Brainstorming, Futures Wheel, and Scenarios. As the study was done before COVID-19, seven researchers of the original group later updated the most likely scenario with new Bibliometrics and Brainstorming. Our findings include that computerization advances will further reduce the demand for low-skill and low-wage jobs; non-standard employment tends to be better regulated; new technologies will allow a transition to a personalized education process; workers will receive knowledge-intensive training, making them more adaptable to new types of jobs; self-employment and entrepreneurship will grow in the global labor market; and universal basic income would not reach its full potential, but income transfer programs will be implemented for the most vulnerable population. Finally, we highlight that this study explores the future of work in 2050 while considering the impact of the COVID-19 pandemic.

## Introduction

Work has been continuously changing throughout history. Industrial revolutions represent “profound changes in the means of production,” and they change work in a short period. We had three industrial revolutions: the first was the implementation of factory-based production using steam-powered machines, the second was characterized by changes in production provided by electricity, and the third was triggered by information and computation technologies [1, 2].

New technologies and their combined use, such as artificial intelligence (AI), robotics, biotechnology, and nanotechnology, are seen as the starting point of the 4th industrial revolution [3]. These technologies are intrinsically associated with socioeconomic changes which, combined, will bring new possibilities for the future of work. Hence, the goal of this study is to use a long-term analysis perspective that considers technological development and socioeconomic changes to explore what work will look like in 2050. The future of work is a challenging topic due to its importance ranging from the global economy to social well-being. Therefore, we hope to help decision-makers from companies, governments, and elsewhere to recognize the changes ahead to better guide our society.

The methodology of this study is based on Foresight. We use four methods from future research—namely Bibliometrics, Brainstorming, Futures Wheel, and Scenarios—to present the main global trends that are most relevant for the future of work. These trends were then further analyzed and consolidated in a likely scenario for work in 2050. According to Grupp and Linstone [4], several countries utilize foresight for policymaking such as EUA, Germany, France, the UK, Spain, Austria, the Republic of Korea [5], Hungary, South Africa, Thailand, Indonesia, Japan [6], Canada [7], India [8], and Brazil [9]. Therefore, this study contributes to the understanding of the current situation of work, and its current future trends—the needed knowledge to perform policymaking changes.

In recognition of the tremendous impact of the pandemic on work [10] and its future, our study was updated with the most recent academic research about the COVID-19 pandemic by new Bibliometrics, Brainstorming, and an update of the Scenarios previously created. However, even before the COVID-19 pandemic, several studies [11, 12] attempted to understand the dynamics behind the future of work and developed sets of scenarios.

## Methodology

This section details the methodology used in this work. First, we present the study dynamics, detailing who participate in the study, how the study was placed in time (number and types of sessions), the foresight methods used, specific goals for each method, and how each method contributed to achieving our main goal, which is to provide scenarios for the future of work in the 2050 horizon. Second, we introduce each method used and explain how they were used in the context of this work.

Foresight studies follow no specific methodology, each study tailor the methodology according to its goals. However, the literature indicates that Foresight becomes more reliable when different and complementary methods are combined [13], once they provide multiple perspectives for the analysis, reducing the probability of a biased result. Therefore, we used a Foresight framework that generalizes Foresights as workflows [14] to structure this study.

In this study, we decided to present our results as scenarios, which is a method to develop consistent evolutions of the future based on a set of assumptions [15] and present the results efficiently to third parties, such as decision-makers. We use the following methods to build the scenarios: Bibliometrics, Brainstorming, and Futures Wheel, respectively. These methods are mostly qualitative; thus, the participants were oriented to base their conclusions on the data gathered in the Bibliometrics method.

### The study dynamic

The study was performed by thirteen researchers (including the moderators) with different backgrounds, for example, in computer engineering, production engineering, public management, architecture and urbanism, and design. These researchers were divided into five groups, taking into consideration their expertise and interests, corresponding to different topics regarding the future of work: computerization/automation (2 researchers), employment (2 researchers), education (3 researchers), social welfare (2 researchers), and economy (2 researchers). The topics were defined by a literature review [16] that was previously done by researchers of the future of work. The other moderator is an expert in future studies. These two moderators participated in all groups, guiding the participants to follow the methodology and providing suggestions as necessary. It is also worth noting that the moderators presented and discussed the results of each step of the methodology in meetings with all the participants of the study. Furthermore, the guidance provided by the moderators and the participation of, at least, two participants for each topic helped to reduce biases and ensured that even though each topic had a relatively small number of researchers. Methods such as Brainstorming and Futures Wheel were performed as group activities, integrating the entire interdisciplinary group of thirteen people in the collaboration efforts to ensure the quality of the results.

The study was conducted during 8 sessions, once per week. Each session had an approximate duration of 2 hours. Since the duration and format of the sessions were defined before the execution of the study, there were few absences from the participants, and most of them were communicated previously. In absences, the moderators explained the work to be done by e-mail and were available to answer any question. Since most of the work was done in the week between the sessions, each group could perform meetings to produce their contributions. However, the Brainstorming for all groups was performed in a single 5-h session with mandatory attendance.

This study was performed with the aid of software, named Tiamat [17]. Tiamat software is a modular collaborative Foresight Support System, designed to support on-site and remote (through the Internet) studies using the concept of Foresight method workflow [17]. The software was used in several studies [12, 18–21] before and allowed the moderators to orchestrate the study, all participants to communicate asynchronously, and serve as a repository that offers traceability to the intermediary results. The Tiamat framework offers a process that can be followed independently from the software; therefore, although we used the computer system to support the study, no special software is required to perform our methodology.

In the first session, the moderators presented the methodology and the Tiamat software and also divided the researchers into the aforementioned five groups. Between the first and the second session, the participants were responsible to search the literature and gather relevant material to be analyzed. The use of bibliometrics is useful to level the knowledge among the interdisciplinary participants, gather and store the state of the art of the topics of the study and select primary citations for further writing future scenarios. Details of the results are presented in the next section.

In the second session, each group presented its findings and the participants had the opportunity to recommend papers to other groups. Each participant would then have two weeks to fully read the papers considered important for their topics.

The third session was focused on discussing their findings until the moment. Each participant would share how the documents that were read up until that session contributed to understanding the future of the topic assigned to their group. Also, the participants were encouraged to share any trends found in the reading that was related to the topic studied by another group, thus stimulating the collaboration between groups.

In the fourth session, the moderators performed five Brainstorming sessions (one for each group) in which the researchers presented the main events they found. For example, the employment group found the event “more flexibility in the employment contract.” In the Brainstorming, we discussed the impacts of each event and proposed new events from the discussion. The Brainstorming ended with voting on which events should be included in the next steps. The moderators lead the brainstorming sessions according to Osborn’s brainstorming guidelines for the generation of ideas [22]. The participants from the other topics were allowed to participate since many papers that they read also discuss other participants’ topics and their different perspectives may contribute to the topic in discussion. The output of the brainstorming is a list of possible future events regarding the topic of the group. These possible future events were used as input for the next method, Futures Wheel. Between the fourth and fifth sessions, the groups were invited to develop a Futures Wheel [23], a method that stimulates participants to discover events that are consequences of other events. The Futures Weel also establishes cause-consequence relationships among events which are highlighted in a graph format. We started the Futures Wheel of each group with the events discovered in the brainstorming as *initial events*, allowing the participants to include, remove, or modify events while they indicate cause-consequence relationships between events, i.e., discovering primary and secondary consequences of events.

In the fifth session, each group presented and discussed their Futures Wheel. At the end of the fifth session, the moderators asked the groups to use the list of expanded events, developed during the Brainstorming and Futures Wheel to identify and develop the main *trends* for each topic using the scenarios technique and the literature already gathered to support their research—new literature could be added further. We call *trend* a set of possible future events that told a cohesive matter, heavily supported by the literature—not only the literature gathered in the bibliometrics step. Each group developed *trends* related to the topic of their study.

In the sixth and seventh sessions, the participants presented the trends for each topic. After the seventh session, the moderators dismissed the topic division, joining all participants to develop 3 scenarios: one which considers the best outcome for each event, one that considers the worst outcome for each event, and one that considers the *most likely* outcome for each event. The participants were asked to check the consistency of each scenario produced.

**Fig. 1:**
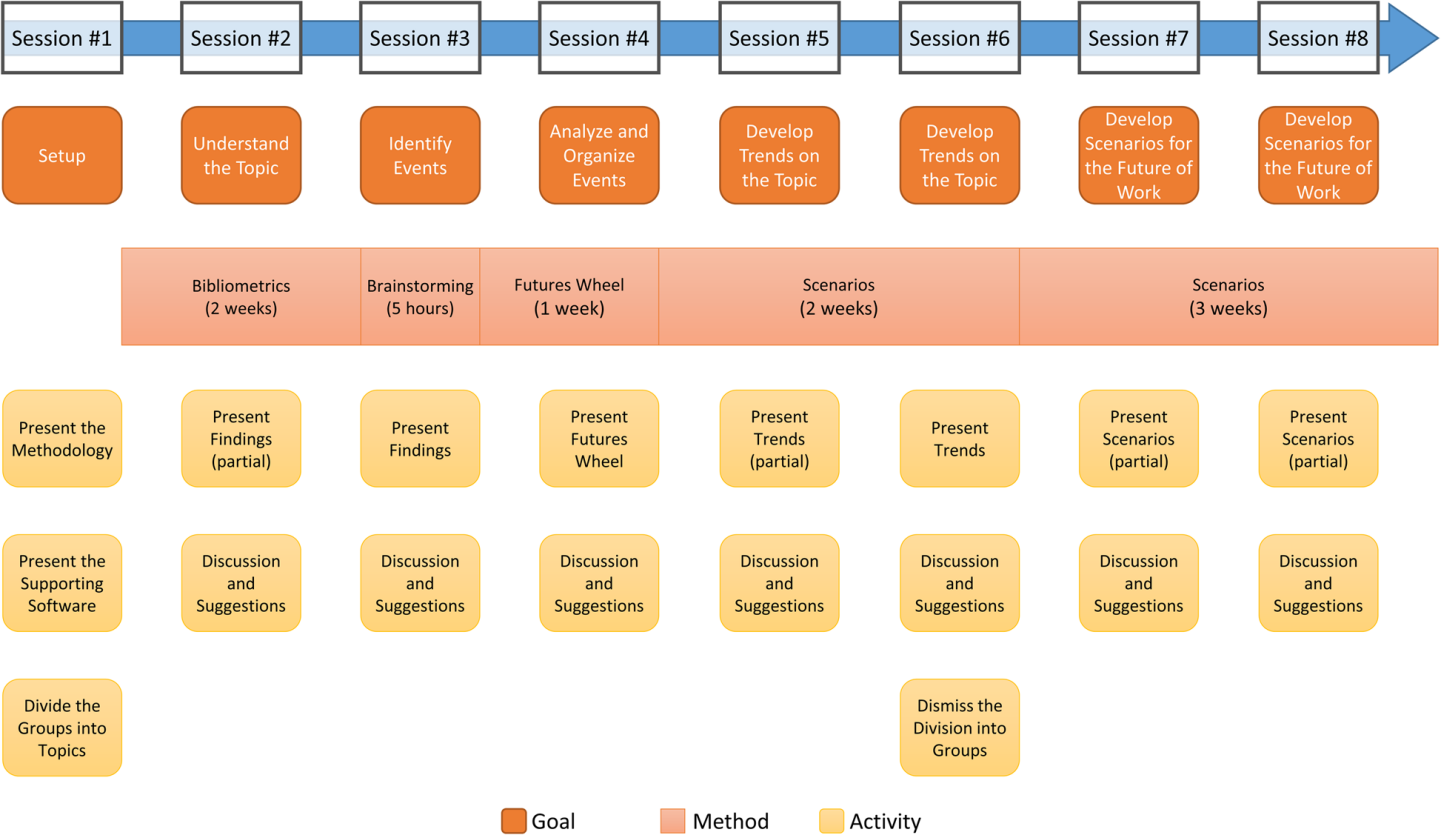
Study dynamic diagram

Therefore, the eighth session was focused to check the produced scenarios. The moderators provided one extra week for the participants to fix all spelling and formatting errors and analyze the proposed suggestions from the eighth session. The final version of the scenarios was delivered using the Tiamat software. This study encounters dynamic is presented in Fig. 1.

With the COVID-19 pandemic, we knew that our study had to be updated to consider its impact. The update was done in 2 months, from June to August 2020, by gathering seven of the researchers involved in the first part of the study and assigning at least one of them to each of the five topics of the research. The methodologies applied to explore the impact of COVID-19 on the future of work and update the trend scenarios were Bibliometrics and Brainstorming. The Bibliometrics was based on 42 papers about the impact of COVID-19 on the different topics that were being studied with several reports serving as a support to grasp the scenario that was unfolding during the pandemic. Then, the group reviewed the likely scenario to consider how the combination of these individual impacts would change the future of work in 2050. We performed the update without face-to-face sessions, as expected during the COVID restrictions.

The resulting trend scenarios presented in Section 3, and the likely scenario presented in Section 4 already consider the COVID-19 pandemic impact on the future of work.

### The methodology in practice

In this section, we formalize the study dynamics: first, we provide a global view of the study, in the form of a workflow; second, we explain how each method work and provide results for methods from one topic as an example of the methodology. The topic of *Employment* will be used to illustrate the methodology in this section. Since trends and scenarios are the main findings presented in detail in this work, we present in this section only example results from Bibliometrics, Brainstorming, and Futures Wheel.

**Fig. 2:**
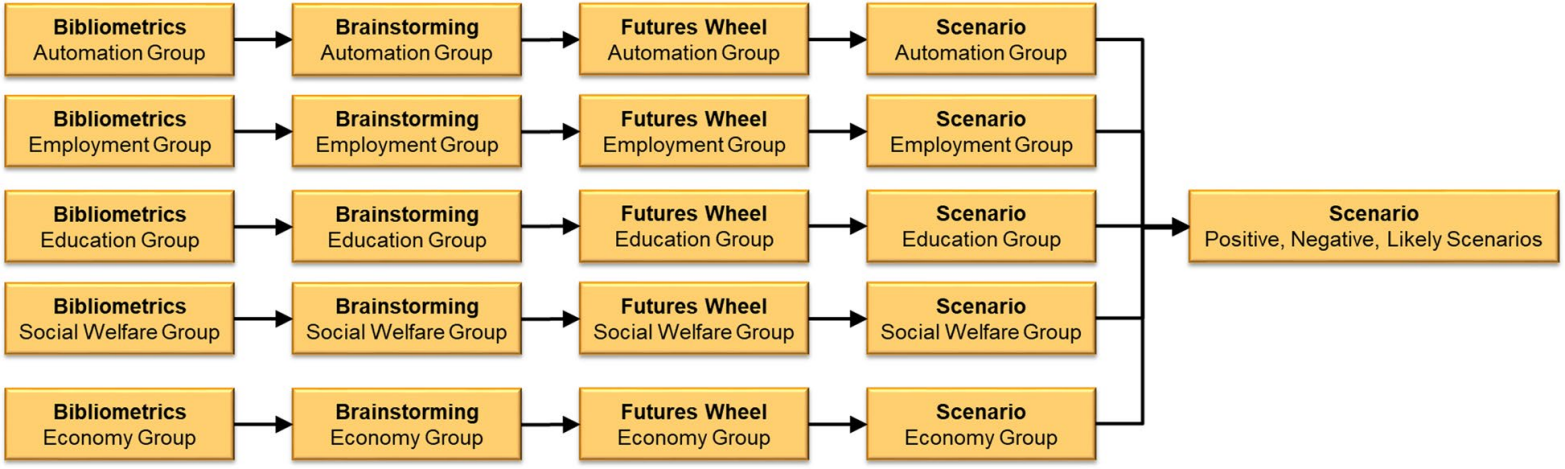
Study workflow

We highlight that the participants were trained and guided during the study to produce high-quality data and guarantee its uniformity and consistency. In Fig. 2, we present our methodology in the form of a workflow following the Foresight framework proposed by Barbosa [14].

The five topics were analyzed individually using four methods: Bibliometrics, Brainstorming, Futures Wheel, and Scenarios.

#### Bibliometrics

Bibliometric analysis is the analysis of large numbers of scientific documents. Bibliometric analysis is usually taken from patent and scientific publication databases [24] and should be combined with other measures or expert opinions to be balanced [25]. The bibliometric analysis summarizes document characteristics for statistical analysis and infers linkage among documents, where it may be used to find indirect links among concepts [26]. For inferring the linkage among documents, there are a few approaches: co-citation analysis, co-word analysis, and mapping. Co-citation increases the linkage between documents as they cite the same references. Co-word increases the linkage between documents as they use the same relevant words. Mapping presents the bibliometric data and findings, facilitating its interpretation by humans.

In this study context, we used bibliometrics to perform a simple literature review, using both scientific databases and documents available on the Internet, such as governmental reports. Therefore, the researchers gathered data in the literature to build a knowledge base, used to identify trends and support scenarios. Moderators did not enforce the use of systematic reviews of the literature; therefore, we infer the use of random search on several bases and Google to include gray literature. Snowballing was also allowed. We expected to reach beyond the academic literature, including data from technical reports from governmental organizations, non-governmental organizations, think tanks, and companies to capture early signals of change and enrich the study by providing plural views about the future of work. Due to the time restriction between the sessions, we back down from performing a mapping of the literature. The summary of the Bibliometrics results is presented in Table 1.

**Table 1:** General results for the Bibliometrics method

Group	Documents gathered
Automation	16
Employment	37
Education	39
Social welfare	21
Economics	19

As an example of an output of the Bibliometrics method, Table 2 presents the results from the *employment group*.

**Table 2:** Results for the bibliometrics method for the *employment* group

Year	Authors	Title
2017	Carl Frey; Michael Osborne	The future of employment: how susceptible are jobs to computerization?
2017	Judy Wajcman	Automation: is it really different this time?
2017	David Tuffley	Can intelligent machines in the workforce lead to a net gain in the number of jobs?
2017	Young Kim; Kyungsoo Kim; SuKyoung Lee	The rise of technological unemployment and its implications on the future macroeconomic landscape
2017	David Spencer	Work in and beyond the second machine age: the politics of production and digital technologies
2016	Ian Herbert; Aravindhan Dhayalan; Andy Scott	The future of professional work-will you be replaced, or will you be sitting next to a robot?
2016	World Economic Forum	The future of jobs: employment, skills and workforce strategy for the fourth industrial revolution
2016	Organization for Economic Co-operation and Development	Future of work in figures
2016	European Comission	The future of work: skills and resilience for a world of change
2016	Chartered Accountants Australia and New Zealand	The future of work: how can we adapt to survive and thrive
2016	Executive Office of the President - USA	Artificial intelligence, automation, and the economy
2016	Andrew McAfee; Erik Brynjolfsson	Human work in the robotic future
2016	Lee Drutman; Yascha Mounk	Will robots kill democracy?
2016	Martin Rhisiart; Eckhard Störmer; Cornelia Daheim	From foresight to impact? The 2030 future of work scenarios
2016	George Strawn	Automation and future unemployment
2016	Keith Cunningham-Parmeter	From Amazon to Uber: defining employment in the modern economy
2016	Geoffrey Hodgson	The future of work in the twenty-first century
2015	Georgenor Filho	Mobilidade Humana e Futuro do Trabalho: Efeitos da Globalização
2015	David H. Autor	Why are there still so many jobs? The history and future of workplace automation
2015	Deloitte	Technology and people the great job-creation machine
2015	Open Society Foundations	Technology and the future of work: the state of the debate
2015	Nick Srnicek; Alex Williams	The future is not working
2015	Charles Petrie	Predictions about the future (of work)
2015	Alan Finkel	Reflecting on the future of work in Australia: pessimism, optimism, and opportunities
2015	Guy Ryder	The International Labor Organization: the next 100 years
2014	UK Comission for Employment and Skills	The future of work: jobs and skills in 2030
2014	Mohammed Qureshi; Rumaiya Syed	The impact of robotics on employment and motivation of employees in the service sector, with special reference to health care
2014	Justine Humphry	Visualizing the future of work: myth, media, and mobilities
2013	John Moravec; George H. Kubik	Limitless: becoming remarkable in the borderless economy
2013	Rob Wilson	Skills anticipation—the future of work and education
2012	Jorgen Mortensen; Marta Vilella-Vila	The future of employment supply and demand in social Europe
2012	Sally Khallash; Martin Kruse	The future of work and work-life balance 2025
2011	Erik Brynjolfsson; Andrew McAfee	Race against the machine: how the digital revolution is irreversibly transforming employment
2011	Kevin Crowston	Lessons from volunteering and free/libre open source software development for the future of work
2011	Ellen Galinsky; Kenneth Matos	The future of work-life fit
2010	Leena Maxin; Jurgen Deller	Activities in retirement: individual experience of silver work
2010	Lynda Gratton	The future of work

#### Brainstorming

Brainstorming [22] is a group technique focused on idea generation that frees its participants from criticism [27]. Osborn’s brainstorming guidelines for the generation of ideas: no immediate concern for quality or evaluation, in a set time frame, encourage building on the ideas of others, and recorded by a non-idea-contributing facilitator/scribe [28]. Although brainstorming is a very old concept, it is still widely used. Putman and Paulus [29] proposed a set of rules based on the original Osborn’s rules but extended for interactive groups. Putman and Paulus [29] proposed rules to avoid criticism, stimulate freewheeling in other participants’ ideas, stimulate quantity over quality of ideas, stimulate the combination and improvement of ideas, avoid losing focus on the task, avoid moments of silence, and stimulate review previously ideas and categories.

In this study context, we used Brainstorming to raise possible future events of each group research topic—computerization/automation, employment, education, social welfare, and economy—on work, based on the literature analyzed in the previous step (Bibliometrics). Moderators place these events are the starting point for the further analysis performed by each research group, following the Putman and Paulus rules. Participants were stimulated to use the literature to develop the events, which were not limited to the Bibliometrics results—i.e, the participants could perform snowballing for example to gather more information. However, the main source of possible future events comes from their understanding of the complex scenario and their further reasoning into ideas. Such ideas—even if they exist—are not easily findable in the literature. Finally, we voted on the list of proposed ideas, developing basic trends to be further analyzed. We present the selected brainstorming events from the *Employment* group in Table 3.

**Table 3:** Results for the brainstorming method for the *employment* group

**Events**
More flexibility in the employment contract
Increased teleworking and working from home
Increased globalization
Job displacement
Technological unemployment
Aging of the workforce
Increased participation of government and individuals in education
Polarization of jobs into low-paying jobs and high-paying jobs

#### Futures wheel

The Futures Wheel [23] is a method to identify the consequences of trends and events. For the sake of simplicity, we will refer to trends or events only as events. Starting initial events, the participants define a set of primary consequences. The participants should ask themselves three questions to discover the consequences: “If this event occurs, then what happens next?”, “What necessarily goes with this event?”, and “What are the impacts or consequences?”. The Futures Wheel analysis continues recursively, i.e., each primary consequence is analyzed to generate a set of secondary consequences. Although the Futures Wheel may go on indefinitely, rarely does it go further than the tertiary consequences, mostly because the complexity of the analysis grows exponentially. Contradictory consequences may also occur and the participants must consider them.

The participants of the Futures Wheel map the event to its consequences, producing concentric graphs, which highlight the potential complexity of interactions, showing that the consequences do not happen all at once, but in an evolutionary, interactive sequence [23].

**Fig. 3:**
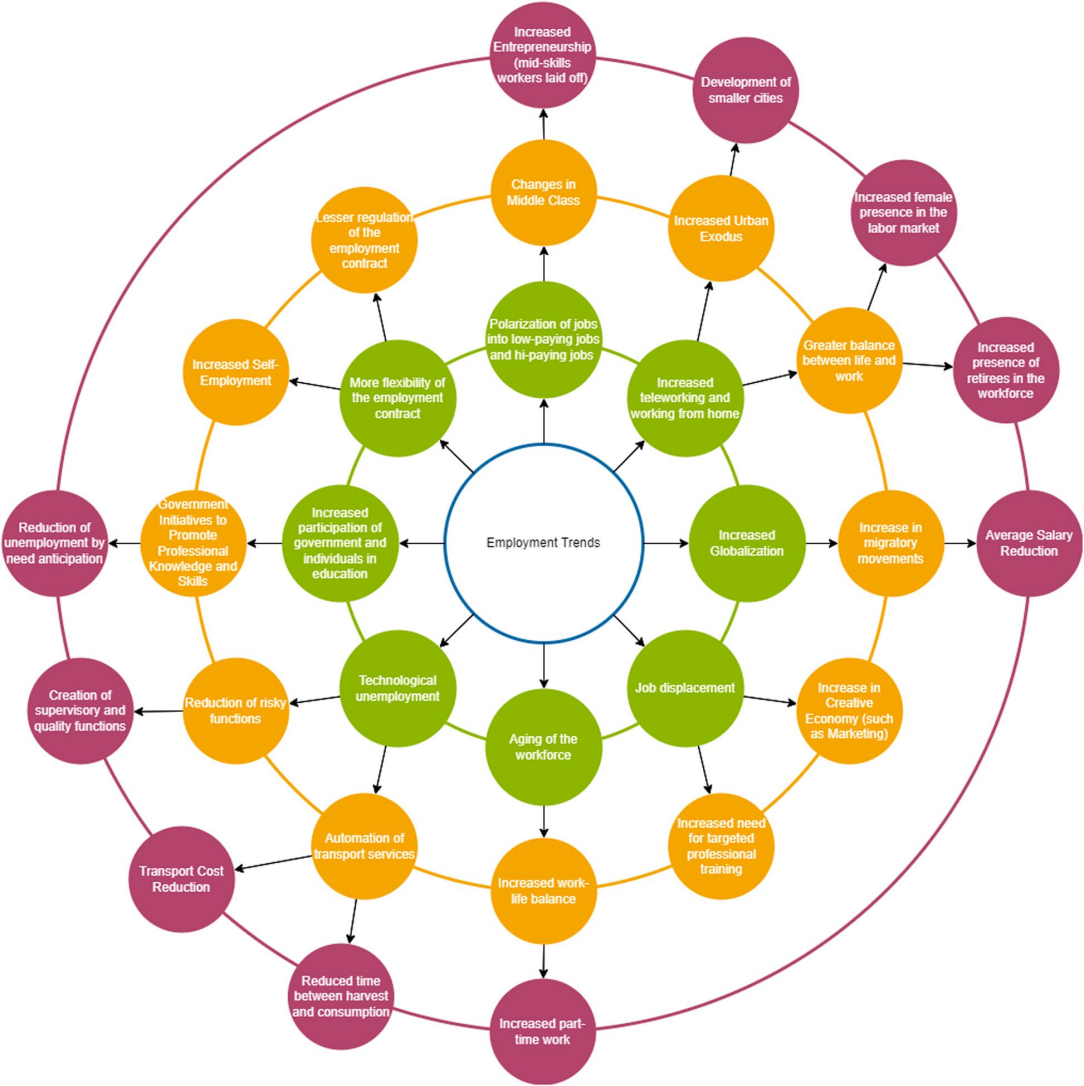
Futures wheel from the *employment* group

In this study context, we used Futures Wheel to further discussed the events listed in the Brainstorms. Therefore, the Futures Wheel mapped the events to their consequences, producing concentric graphs of primary, secondary, and tertiary consequences. New events were included as a result of this analysis. The Futures Wheel from the *Employment* group is shown in Fig. 3.

#### Scenarios

Scenarios are possible evolutions of the future consistent with some set of assumptions [15]. Scenarios have been termed the “archetypal product of futures studies” [30]. They can be achieved through creative thinking about future possibilities (explorative scenarios) as well as through active working towards the production of a desirable future or set of futures (normative scenarios) [31].

Scenarios represent the combination of a set of extrapolated current trends or projections, and these must be internally consistent, i.e., not contradict each other. For example, when analyzing possible futures related to ATM usage, a scenario where an increase in cashless money transfer and an increase in the usage of ATMs by the general population should be pruned, as these events are mutually exclusive, therefore making the scenario inconsistent [32]. Indeed, Shoemaker [33] suggests that three tests of internal consistency are especially useful. Firstly, remove scenarios with trends whose time frames do not match. Secondly, remove scenarios in which predicted outcomes are inconsistent with each other. Lastly, remove scenarios in which major players are placed in unlikely positions.

In this study context, we used scenarios to analyze the events and developed the trend scenarios that are presented in detail in Section 3, using each group Futures Wheel and literature gathered. Therefore, the trend scenarios discuss trends for each topic of this study, and they are heavily based on the literature.

Finally, we also use scenarios to develop three scenarios for work in 2050: an optimistic/positive scenario, a pessimist/negative scenario, and a likely scenario. To develop such scenarios, we dismissed the division of groups into topics, since the scenarios must consider all topics. The Scenarios for work in 2050 were built on all the knowledge gathered in all previous steps. Therefore, the scenarios are based on the joint analysis of the trend scenarios to understand how they interact. We also classify the trends as more or less likely to happen and if a trend can be considered *good* or *bad* for society. Due to space limitations, we present the likely scenario in Section 4, which considers the combination of the trends for the future of work that the participants considered as *most probable*.

#### Trend scenarios for future work

This section will present the future trends for the areas analyzed in this study: computerization/automation, employment, education, social welfare, and economics.

### Computerization/automation

The last century started a transition in industrial automation as machines are increasingly better to make decisions, not only performing manual activities but allowing more activities to be automated. The most cited paper concerning the topic estimated that 47% of the US workforce was under a high probability of computerization (automation by computer technologies, mainly AI and Robotics) in the next decades [34]. Later studies that applied the same methodology showed that the number of workers in occupations that are likely to suffer computerization varies from country to country. In developing economies such as Brazil, the percentage reaches 60% [35] while in advanced economies such as the UK, the number drops to 35% [36].

Areas such as the retail market, archiving, data collection and processing, and line assembly operations will be highly impacted. Still, even for workers at higher risk, adopting automation is not simple: it requires analysis of some key points, such as technical feasibility; development and implementation costs; labor market dynamics, considering its demand, costs, and social characteristics; economic benefits, such as governmental policies; and social acceptance [37].

As automation increases, it will require policies to protect unprepared and vulnerable workers, allowing them to migrate to the new model of production [38]. Underdeveloped nations face higher risks since they are rarely part of the discussion about this topic and are outside of the focus of studies. Erroneous interventions also leave underdeveloped nations incapable to compete against developed nations, producing economic, social, and political inequalities along with technological advancement [38]. It is important to note, however, that unemployment levels have remained stable in the long run, despite disruptions caused by industrial revolutions, as workers migrated to new jobs sometimes enabled by new technologies or the number of jobs was increased because of a higher consumption [39, 40].

The increasing adoption of automation technologies results in ever-lower costs of hardware, sensors, network, processing, and storage; a more refined and accurate set of data allowing tests and studies even without human supervision; and a great expansion and absorption of knowledge unprecedented [41].

The last 20 years have brought remarkable progress in AI, one of the most important technologies in the current wave of automation, and now, we can build machines capable of learning even when humans are unable to teach them, producing new knowledge faster than humans [41]. Due to these advances, it will be possible to have AI working with humans as assistants, from reading e-mails to driving cars. However, it will also raise privacy, security, and ethical issues, with unintended consequences if we cannot identify these challenges promptly [42].

The Internet of Things (IoT) is another important automation technology that has been experiencing considerable expansion, especially in areas such as medical and health care, smart building, intelligent transportation, industry, and logistics [43]. IoT includes low-cost and high-performance processors attached to low-cost sensors; they usually include some form of analytical software, many of them in highly distributed architectures, i.e., cloud computing [44]. IoT is a central element of Industry 4.0 where the integration between humans and machines can speed up the production systems by 30%, raising their efficiency by 25%, and allowing a new scale of product customization [45].

IoT evolved into the development of smart medical devices creating the concept of the Internet of Medical Things (IoMT). IoMT allows real-time monitoring of the health condition of a person using smart sensors and connected devices and can also help the medical staff at the hospitals by remote monitoring chronic-conditions patients at home. Thus, IoT reduces the workload on medical staff and becomes a necessity instead of a luxury [46].

The COVID-19 pandemic affects several automation-related segments such as telemedicine, IoMT, manufacturing, and supply networks, AI, and smart payments. In telemedicine, the increased adoption of telemedicine to keep patients and medical staff safe can be highlighted. One example is Tele-Critical Care (TCC), a tool related to telemedicine that enables intensivists in traditional intensive care units (ICU) to speed up critically ill triage, thus improving ICU bed management; in hospitals without ICU, TCC enables remote care for critically ill patients, preventing transferring these patients. Another telemedicine tool is Telementoring, in which experts from low-demand areas help their high-demanded peers. During the COVID-19 crisis, intensivists used tiered telementoring to provide consultation on patients with a higher risk from experiencing respiratory and organ failure. One intensivist was capable to oversee 100–250 patients through telementoring [47].

Manufacturing is also being adapted for the post-COVID era, as workplace standard practices are adapted to the physical-distancing policy, thus stimulating concepts such as Smart Manufacturing and Industry 4.0 [48, 49]. AI will be the backbone of automated transportation systems, both on the streets (driverless trucks and cars), and in factories and warehouses (Automated Guided Vehicles) [49]. AI is related to the development of cleaning and disinfecting robots as well [46]. Such innovations make manufacturing and supply chains more resilient to human-related interruptions.

Finally, smart payment (also known as contactless payment) technologies minimize human contact during cash payments; their demand tends to continue high in the post-COVID era [46].

The COVID-19 pandemic accelerated the adoption of several technologies—such as Big Data, robotics, AI, and IoT—as they help companies and society in general to mitigate the impacts of the pandemic. In some cases, the adoption of technologies was necessary to maintain the operation of businesses, making digital literacy an essential skill, and allowing workers to see technologies more as a tool than a replacement [50], a movement that started even before the pandemic as digital skills were already becoming an important determinant of employability in the digital age [51, 52]. The adoption of automation and digitalization tends to intensify during and after the pandemic as essential activities will use more automation to safely attend to their customers and activities that can be moved online such as retail, entertainment, and recreation will be digitalized [53].

### Employment

The world is entering its 4th Industrial Revolution where technologies such as AI, nanotechnology, 3D printing, robotics, and biotechnology are being used in combination and creating new possibilities for production [54]. Technological unemployment is once again a preoccupation in this new industrial revolution and it can be defined as “non-employment due to our discovery of ways of saving the use of labor, exceeding the pace at which we can find new uses for work” [55]. On the other hand, the displacement theory of work affirms that automation will provoke the end of certain careers and the creation of new ones, thus causing little or no harm to employment.

Globalization, another major force in the future of employment, has created two trends in the markets: outsourcing and immigration. Remote work is already a reality, even in traditional enterprises such as IBM, where only 42% of employees work in IBM’s location [56]. Remote work was only recently adopted by large companies, but in startups, it is already common. The distribution of offices in different places or even spaces for co-work will promote a reduction of expenses for the companies, becoming an alternative to the central offices in costly commercial locations [57]. However, illegal immigration from underdeveloped nations will be motivated by the combination of unemployment, food scarcity, wars, and other extreme situations [58].

There is also a trend for greater flexibility for workers, making it possible for them to mix different part-time jobs. In this scenario, virtual reality (VR) and augmented reality (AR) may be used to amplify immersion and collaboration, allowing workers to be “where” they are needed [59].

The return of the elderly to the workforce will be motivated by the increased difficulty in fulfilling their retirement plans, in general, and also by the sense of helping society with their experience [60, 61]. This trend produces a significant impact on society since organizations can continue to be competitive by having access to a larger pool of qualified professionals, reducing the scarcity of specialists, and the impact on social security systems [60]. Considering advances in the automation of health care, increased use of continuous health tracking devices, reduced health costs, and human errors being reduced due to automation, life expectancy, and the time that a person will be able to perform work and actively participate in society tend to increase [62, 63].

According to the International Labor Organization (ILO) [64], “non-standard forms of employment have become a contemporary feature of labor markets around the world.” In South America, 6 of the 10 young people working in the informal economy today [65]. This trend is not exclusive to developing countries [66].

Recognizing the inevitable growth of non-standard forms of employment, a policy proposed by Harris and Krueger [67] introduces a new “self-employed” designation that is not eligible for overtime payment and unemployment insurance but protects workers by antidiscrimination statutes and gives them the right to organize and withhold taxes [67]. Their employers, be they online or offline, would make tax contributions to the payroll [67].

The labor movement suffered recent changes influenced by globalization and technological change but has managed to remain relevant as new forms of work and challenges for workers appeared [68]. Some examples of how the labor movement is being organized by digital platforms’ workers are the App-Based Driver Association, a group from Seattle-US of app-based (e.g., Lyft, and Uber) drivers, and Turkopticon, an initiative by the University of California San Diego that gives the possibility of Amazon Mechanical Turk workers to evaluate their Human Intelligent Tasks [69]. Another way that new labor movements can be created and empowered is by seeking support from traditional unions and other social actors. An example is the FairCrowdWork Watch, a platform developed by the IG Metall (dominant metalworkers’ union in Germany)—that allows workers to rate platforms, compare their payments with others, and receive legal advisory [70]. The diversity of new workers’ movements and the importance of their agenda show that these organizations are likely to continue existing in the future by adapting themselves to each new challenge with the support of technology. Nevertheless, this trend does not mean that traditional unions will become more relevant in the future as digital platform workers tend to feel a certain apathy towards unions partly explained by their identification with entrepreneurship [68].

The job losses caused by the current pandemic are expected to be worse than the 2008 crisis because around 38% of the global workforce is in economic sectors that are suffering a collapse in demand such as manufacturing, hospitality, tourism, and transportation. The crisis is also expected to increase unemployment rates around the world to two-digit numbers even in places that had very low rates before the pandemic as the USA and developing countries can experience even worse outcomes [53].

In Italy, an analysis of the impact of the coronavirus on 7800 companies shows that the aggregate shock on a 3-month horizon is −21% and −16% in twelve months with companies canceling 44% of the preexisting scheduled R&D plans. When it comes to employment, the expected aggregate drop is −6.5% [71]. In the USA, a survey of 10,000 households shows the impact of the virus from January to April of this year. The employment rate fell by 5%; overall spending dropped by US$1000 per month (a 31% drop), especially with mortgages, student, and auto loans which indicates the possibility of a wave of defaults soon; 42% of employed respondents lost earnings due to the virus (an average of over US$5000) [72].

Another challenge brought by the pandemic is the asymmetry of the impact on jobs as it will disproportionately affect entire social categories as low-skilled, low-wage jobs usually held by minorities, immigrants, women, and other disadvantaged groups will suffer the effects of the crisis in the long term [50, 53].

COVID-19 showed the importance of the low-wage workforce which comprises a considerable portion of the essential sectors. Immigration systems in advanced economies tend to be more open to high-skilled workers and more restrictive when it comes to low-skilled workers. In the UK, 16.1% of essential workers are foreign-born. Specifically in the health industry, 18.6% of the workforce is foreign and 13.4% of the workforce is not from the European Union. In a “sharp” crisis like the one caused by COVID-19, immigration systems cannot change quickly enough to supply immigrant workers to needed areas. Forty-six percent of foreign-born essential workers in the UK do not meet the post-Brexit immigration rules that stipulate minimum thresholds for immigrants’ jobs’ skills and wages [73].

Many unemployed people will seek jobs in other cities and countries, leading to migration. Massive migrations to other countries may cause two impacts: anti-immigration laws in the countries receiving immigrants and a lack of young workers to develop the countries losing their workforce [74]. According to Goniewicz et al. [75], future policy should incorporate lessons learned from the COVID-19 pandemic. Granting refugee status to immigrants is controversial and pro-immigration policies can cause confusion and conflicts [74]. A policy for long-term social distancing and the gradual personal interactions of low-risk individuals should be implemented. Workplaces should be adapted to facilitate physical distancing.

At the micro-level, as the measures to control the coronavirus spread involve social distancing, society is experiencing a surge in remote work, specifically working from home [76]. This change brings new challenges to workers as unplugging from work demands is one of the new work-life conflicts [50, 76–78].

### Education

The changes in the world of work will force education to be adapted and advancements in technology may help teachers to achieve this goal. The education system needs to train increasingly specialized workers, due to the end of some careers and the emergency of new ones [79]. This cycle will be more active and impactful in the future, bringing the need for lifelong learning to adapt workers to different jobs. However, the reactive characteristic of changes in education (that trains workers for an almost obsolete job to bring them to the current market) needs to be adapted, continuously updating their curricula on new job trends, and providing relevant competencies for job opportunities [79]. Governments and education institutions have a key role in keeping education updated for new workers. Governments are also responsible for stimulating the creation of new jobs. In this way, they create initiatives such as short-term higher education courses focused on a faster insertion into the labor market [80] and Massive Open Online Courses (MOOC) capable of teaching and training thousands of workers at the same time, complementing traditional educational methods to provide faster adaptation of education in the future [81].

As work will need less time to be performed due to automation [79], workers will have more free time, which may be used to learn, rest, or work on a second job. Information becomes cheaper (in many cases free), brought by the expansion of knowledge through the Internet, and this trend boosts the use of MOOCs by workers. Thus, we will see teachers becoming advisors, directing students through the knowledge freely available [79]. Education will be personalized, with tailored learning plans to fulfill the worker’s needs, interests, and preferences stimulating students to spend more time learning the skills related to their interests [82]. Besides, projects such as the open access initiative will help the sharing of research facilitating free access to knowledge [83]. MOOCs and other educational online environments have also the capability to train people looking for self-employment, either to supplement their monthly income or as the only existing employment option.

New jobs will require highly skilled, knowledge-intensive workers [84]. Science, Technology, Engineering, and Mathematics (STEM) skills play a key role in any country’s economic success and require years of investment in education [85]. This higher demand for highly skilled workers will also affect low-skill jobs, mainly those in services [85]. Fundamental skills such as literacy, numeracy, communication, and team working are required for most jobs [85, 86].

In addition, the worker of the future will need to learn the following skills: critical thinking and problem-solving (cognitive skills), presentation and conflict resolution (interpersonal skills), and adaptability and self-development (intrapersonal skills) [87]. Jobs with a lower risk of automation rely on social and creative skills [84]. Therefore, the most important skills needed for these jobs are collaboration, self-regulation, knowledge construction, communication, real-world problem-solving, and the use of technology for learning [88].

During the COVID-19 pandemic, millions of students were unable to go to school and received general recommendations to use digital tools such as online study platforms [89–91].

Before the COVID-19 pandemic, we assessed that as free information becomes more prevalent in society, we may see teachers replaced by MOOCs if people became more self-taught. From mid-March to mid-May 2020, Coursera, one of the most used MOOC platforms, saw a growth of ten million new users [92].

Regarding the impact on schools in the USA, Dutta [93] discusses several consequences of prolonged stay-at-home and school closures for children. Prolonged isolation from their grandparents, teachers, classmates, and friends is likely to cause sadness and stress. The unexpected transition to distance learning makes students struggle to learn the required knowledge for their respective grades, while schools face difficulties with the standardized COVID-19 test requirements. Schools also play an integral role in promoting healthy eating and maintaining an active and healthy lifestyle—which may lead to sedentary behaviors and, thus, increased rates of child obesity. Inequality is also an issue: some students will have problems accessing the distance learning web modules, and children in need will lose access to school meals, facing starvation. Kneale et al. [94] list other consequences: children losing access to school-based health care, increased injury risks due to self-care or inadequate care, higher risk of child abuse or violence, and even increased child labor and marriage rates.

The World Bank produced a report on the impact of the pandemic on education financing. They projected declines in government revenue due to slower economic activity. On the other hand, countries are overspending their budgets on health and social protection. Such a combination will deteriorate the fiscal balances in most countries during 2020. Therefore, governments will tend to reprioritize their budgets, reducing the education budget, reducing the per capita education spending in almost all country income groups and regions, and impacting future education outcomes. Even in a scenario of economic growth for 2021, the education budget tends to stay stagnate or fall in most countries [95].

### Social welfare

Technological progress will affect how we work. In healthcare, technology will increase the quality of diagnoses and improve people’s quality of life. The population is proportionately aging [96], and, as a result, people’s productive ages will increase, raising the economically active population. Besides, a bigger population implies changing pension systems as well as workers who exceed the minimum/normal retirement age for a better pension.

In general, eligibility rules for retiring are complex and the pension benefit varies according to the objectives of each government. In 2014, the normal age of the normal pension was 64.0 years (men)/63.1 years (women), assuming entry into the labor market at age 20 [97]. The forecast for 2054 is a rise in the normal retirement age, with more countries raising the normal retirement age to above 65 years while reducing the gender disparities in retirement age [97].

**Fig. 4:**
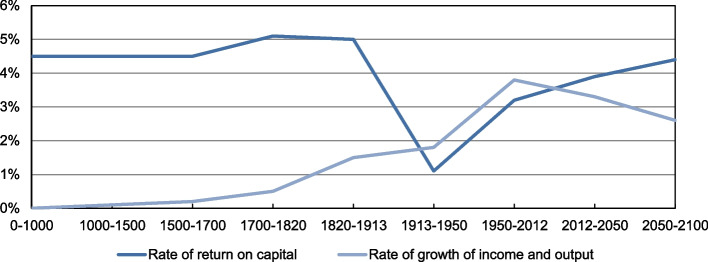
After-tax rate of return on capital vs. growth rate at the world level, until 2100 [98]

Figure 4 shows a forecast that economic inequality tends to grow in the future, bringing negative consequences for the distribution of wealth, since previously accumulated wealth grows faster than production and wages. Thus, the current wealthy people tend to become the dominant rentiers over those who do not own properties but only their work [98].

Gender equality influences the return on capital rate and the growth of income and output. An increase in women’s participation in the economy results in more political power. If the trend toward increasing gender equality is sustained in the coming decades, a slower increase in economic inequality can be expected [99].

Gender equality has improved in the last 50 years, but several countries still fail to provide even fundamental rights to women, especially in North Africa and the Middle East. The gender gap has slowly reduced in the past decade. Health and education subindexes reached values close to 1 (equality), while economic and political show much lower values. At the current rate, the gender gap will only be closed by the year 2100 [100].

Some researchers argue that growing inequality is a result of the exponential shift in technology. The new technology provides the economic reward for the winners of our modern economy, while the losers become increasingly expendable and less resourceful [101]. Racial inequality should be also considered. For example, Native Americans, Africans, and Latin Americans present a lower Human Development Index (IDH) than Asian and white Americans [102]. Sexual orientation is also a taboo subject in many countries, making it difficult to develop evidence-based policies. LGBTI rights are also necessary for an equal society. Anti-discrimination laws are necessary to make people more tolerant of the LGBTI community.

For McGahey [103], technology or computerization job losses, demographic changes, and rising costs of social benefits are challenges for social welfare states. Thus, social welfare states should offer new types of social benefits or build a new way out of this problem. McGahey suggests that there is a way for introducing Universal Basic Income (UBI) as a floor income, providing basic subsistence, complementing the existing welfare state policies, or, in some cases, substituting it [103]. Universal Basic Assets (UBA), sometimes considered an evolution of UBI [104], is defined as a basic set of resources every person is entitled to have: housing, education, health, and financial security.

The COVID-19 pandemic affected social welfare in several ways because it causes an economic crisis and global recession. Although each country tries to stimulate its economy, methods vary due to financial and political constraints. US unemployment raised to its highest level since the great depression. Most households have insufficient savings to live through this type of adversity. Governments provided liquidity to the most vulnerable households through penalty-free withdrawals from retirement savings accounts and stimulus checks, among others. The consequences of such actions include large government debts [105].

Massive unemployment led many families with young children to become food insecure. Household Food Insecurity (HFI) increases the risk of chronic undernutrition and infectious diseases in children, maternal anemia, obesity, and type 2 diabetes [106]. According to Pérez-Escamilla et al. [106], COVID-19 HFI will affect more vulnerable groups such as young children, pregnant, and lactating women. Many disadvantaged students lost their access to free school meals [74]. Half of them are in low- and lower-middle-income countries; losing this meal also reduces the most vulnerable families’ income [106]. Under such circumstances, children in some nations are at higher risk of child marriage and child labor [74]. COVID-19-related stockpiling and panic buying have also affected food security. The just-in-time supply chains are vulnerable to disruptions; this sudden rise in demand caused empty shelves and higher prices for some products. Poor availability of food in supermarkets forced households to access food from food banks that already suffering from the sudden increase in demand and reduced volunteer numbers. Some independent food banks achieved their “breaking point” and others closed entirely [107]. According to Power et al. [107], the food aid system seems unable to face health and economic emergencies simultaneously. According to Pérez-Escamilla et al. [106], COVID-19 has shown “how unprepared the world is to protect populations against hunger, food, nutrition, and health insecurity during global emergency situations.”

### Economy

The current demographic changes will highly impact the economy in 2050. The first demographic change will be caused by world population growth, which will rise from the current 7.6 billion to about 10 billion people by 2050 [108]. This worldwide increase in population is a challenge as it shows that hundreds of millions (or a few billion) jobs must be created [108].

A second demographic change that will impact economies is population aging. By 2050, the population of developing countries will still be younger than those of developed countries [109]. As the worker live longer, they must also have to work for a longer period to support their pension schemes. The extension of working years will also impact youth employment as more experienced workers will dispute a few job opportunities with them.

A third demographic change is an increase in urbanization. Besides the population growth, people in rural areas are migrating to medium and large cities. According to Schwettmann [108], 64% of the population of developing countries and 86% of the population of developed countries will be urbanized by 2050. This trend has mixed impacts, as urbanization may cause unplanned city growth, pollution-related health hazards, and unemployment. However, urbanization may reduce the costs of transport and education, and create cultural diversity [108].

The 2050 economy will be based on knowledge-intensive work. Knowledge-intensive services now include business services such as finances, accounting and software, medical services, and engineering [110]. Knowledge-intensive careers have fewer jobs; mostly because they require very high skills and advanced degrees in the fields of science and engineering. Thus, the unskilled and less educated, which represent most of the population of many countries, are excluded from most of the opportunities in knowledge-intensive production [110].

Technology is only a factor to determine economic results such as growth, inequality, or employment—however, technology is the main driver of Gross Domestic Product (GPD) growth per capita. Leading economies have access to similar technologies which resulted in different economic results throughout history, mostly because they have different policies and institutions [111].

According to the McKinsey Global Institute [37], in the USA, 46% of the time spent on work activities is technically automatable, using the current technologies. They estimate that the current automation technologies could replace 50% of working hours on a global scale. Increasing computerization will affect almost every occupation, not limited to factory workers. This automation potential represents 1.2 billion workers, which wages about US$ 14.6 trillion [37].

Rises in labor productivity usually translate into increased average wages, providing the opportunity for workers to reduce their working hours and increase the offer of goods and services [111].

**Fig. 5:**
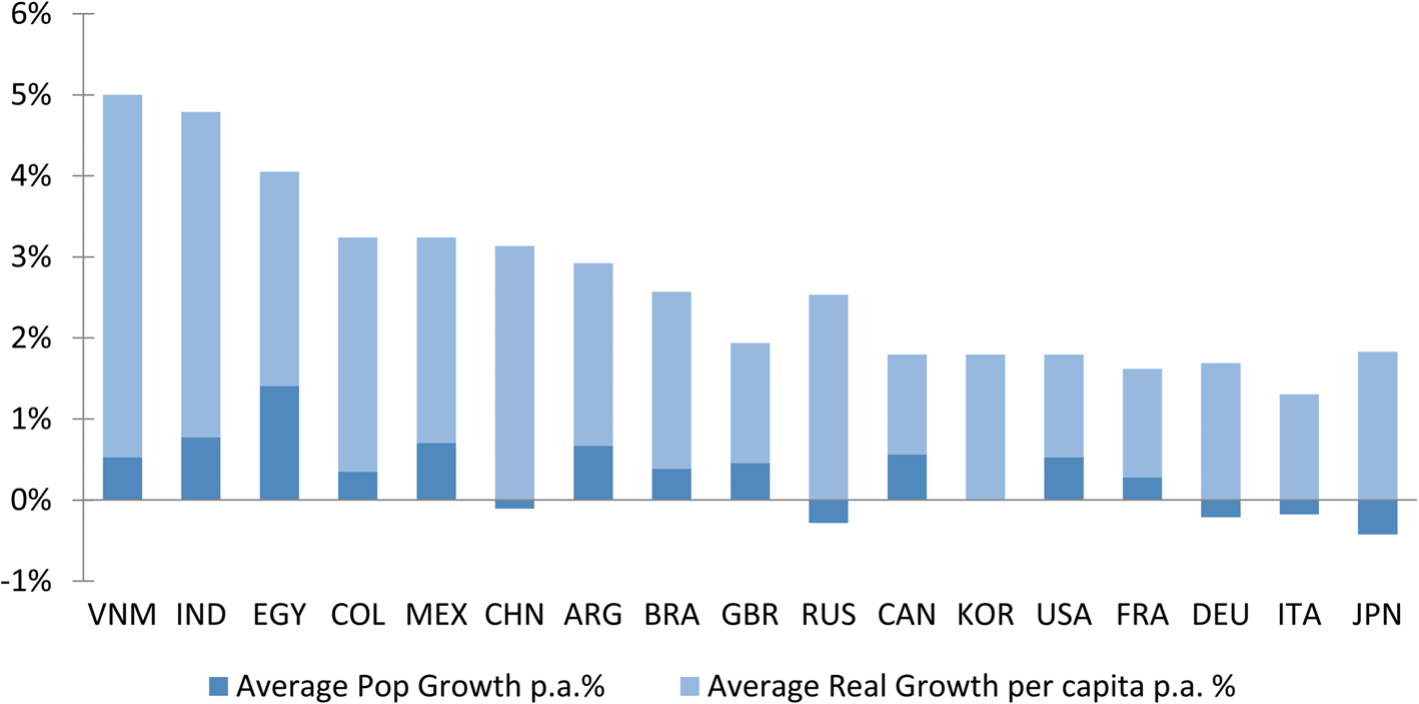
Projected average real GDP growth 2016–2050 [109]

Another important trend related to the economy is a phenomenon named “Rise of The Rest,” which describes the shift of the GDP from developed countries to developing countries. Nowadays, global economic activity is already shifting from the G7 to the G20 [109, 112]. As a consequence, developing countries have a faster increase in their technology, capital, and people [109, 112]. Figure 5 presents the projected average GDP growth from 2016 to 2050. We highlight that COVID-19 and the recent Russian invasion of Ukraine [113, 114] may cause an impact on these long-term GDP projections, especially for Russia.

The COVID-19 pandemic produced a health and economic crisis with unprecedented scale and magnitude producing an unforeseen combination of supply and demand shocks for the global economy that will affect it for a long time even after the coronavirus control policies [71]. Almost 90% of the world economy went under some sort of lockdown measures by mid-April and an economic crisis unparalleled to none since the great depression has taken place. Blockades of national frontiers imposed by governments have paralyzed economic activities in general, laying off millions of workers worldwide and having a major impact on the world economy. Global GDP is forecast to decline by 3.2%, reaching a drop of 5.0% among developed countries; and production losses projected for 2020 and 2021—almost US$ 8.5 trillion—will eliminate almost all production gains of the previous 4 years [53, 115, 116].

Among developing countries, large fiscal deficits and high levels of public debt will pose significant challenges, particularly for economies dependent on commodities and tourism. The severity of the economic impact depends mainly on two factors: the duration of the restrictions (economy, circulation, and transport) and the size and effectiveness of fiscal responses to the crisis [117].

Households are strongly affected due to lockdown restrictions, causing job losses. This situation reflects in a decreased consumer power, being perceived as more accentuated in sectors such as tourism/hospitality and clothing [72]. The greatest concern on financial health at the family level is due to the uncertainty as to whether financial reserves will cope with an extended lockdown period [117]. For this reason, governments have introduced financial support programs for groups of people who are economically vulnerable to the effects of the pandemic; however, not all have established norms regarding credit scores, which also have an influence on consumption and the granting of future credit [118].

The global production chain was also impacted: mainly affected by the closure of countries’ borders, this network of international economic relations proved to be highly dependent on a small number of countries, such as China—causing the absence of industrial inputs and unbalancing the trade balance of the countries under its influence [117].

The COVID-19 pandemic also impacts the financial sector. Risk management models built over the past few decades have not been able to guarantee global financial stability and contain the effects of this financial crisis [119]. Stock exchanges are experiencing a period of high volatility around the world, mainly in Asia—investor uncertainty reflects the pandemic’s effect on the economy [120].

The governmental response involves a set of strategic actions, learning lessons from this event to build resilience for possible future crises. At the household level, governmental actions include proposals for financial support for those who had income losses and for stimulating family cost savings to build emergency finance reserves. At the business level, governmental actions include preventing corporate bankruptcy and mass layoffs by identifying companies that are in the most critical stage to support loans and investments so that they can rebuild. At the local economy level, governmental actions include identifying interventions to improve business recovery after COVID-19 and prioritizing investment in critical economic sectors and businesses, based on the added value to the local community [121].

Stronger development cooperation, supporting efforts to contain the pandemic, and extending economic and financial assistance to countries most affected by the crisis will be of utmost importance to accelerate the recovery and put the world back on the path of sustainable development [115].

### Likely scenario for work in 2050

In this section, we use the trends and trend scenarios presented in Section 3 to build a most-likely scenario of how working will be in the year 2050. First, a short story of this scenario is told, followed by a discussion of the actions that the social actors (government, companies, and workers) would take to lead us into this future.

### Working in 2050

The advancement of computerization will make some jobs obsolete while new ones will be created, as happened in previous industrial revolutions. The benefits generated by automation are important and induce widespread improvements in society as society has taken the right actions to guarantee that technology adoption does so, at least in most cases.

Occupations that involve social and creative intelligence, and/or advanced perception and manipulation tasks will be less affected by computerization due to the technology limitation to emulate these behaviors.

Society will see a job shift from low-skill to knowledge-intensive occupations. Many people will face unemployment and companies will be stimulated by governments to help reduce the transition impact by training workers in new skills before completely automating their jobs. Those that cannot be helped by these measures will receive a basic income from the state.

Communication will be globally improved due to the reduction of costs in Internet access in most countries. Better communication will further improve the integration between national and international markets, allowing more people to offer their services on the internet. The COVID-19 pandemic and the measures adopted to contain it still have an impact on work because by 2050 employers will be aware that they may be forced to move their production to remote work at any time. Thus, we expect some jobs to have at least one “remote day.”

Workers will not be associated with traditional trade unions as new forms of worker organization movements will be recognized by governments and employers. These organizations will show that new technologies can be used to innovate not only the way people work but also how they defend their rights as workers.

As populations age, the minimum retirement age will increase. This will not represent a big impact on companies for, as jobs become more knowledge-intensive, it will be increasingly easier and even profitable to keep senior workers in their jobs.

Gender equality will increase, but even in 2050, many countries will still be far from the ideal equality between men and women. Another group that will see advancements in their rights, despite some resistance from far-right groups, will be the LGBTQIA+ as more initiatives such as anti-discrimination laws are created. Racial discrimination will also reduce.

The income distribution will be another factor for social inequality reduction. Several UBI and UBA trials will happen worldwide, but few societies are going to adopt these social welfare policies permanently.

The world population will be close to 10 billion, and governments will face the challenge of creating jobs for hundreds of millions or, at least, providing means for their survival. As some countries will fail to do so, workers will either migrate or use the internet to offer their services globally. Both developing and developed countries will see a reduction in their rural population as urbanization grows.

In 2050, the economy will be more knowledge-intensive as low-skilled jobs are reduced and new ones, based on more creative and social activities, are created. Rises in labor productivity will provide increased average wages, allowing workers to reduce their working hours while the offer of goods and services is increased.

The COVID-19 crisis will affect the global production chain in the long term. Many governments will consider the production of some fundamental industrial inputs as strategic, demanding local production by law, even with higher costs. This will reduce their dependence on other countries.

Several countries will face large fiscal deficits and high levels of public debt, aggravated by the COVID-19 pandemic. The severity of the economic impact will be related to how strongly each government acted to break the spreading of the SARS-CoV-2 virus. Another effect to be seen is the increased migration in the search for better economic opportunities, which will lead to an increase in nationalist and far-right movements in Europe and the USA.

### Actions leading to the most-likely scenario

In this section, we present a compilation of a set of actions that could be taken to make the most-likely scenario come to fruition. Most of these actions are related to public policy and have been mapped during the different steps of the research.

For the most-likely scenario to become true, governments will have to make sure that technology meets not only the capital demands but also the population’s needs. As such, governments that promote regulatory policies to control the advancement of computerization, avoiding mass unemployment without stopping innovation, will perceive better results—both in economic growth and welfare. These regulatory policies include the ones aimed to protect vulnerable people, those with few resources and less educated, who must be prepared for this transformation. Governments will be able to respond to the job shift by investing in broader and better education and teaching new skills for the new occupations that will arise while providing the displaced workers with, at least, the minimum living conditions during this period of transition [122].

As companies perceive the consumer market reduction risk due to unemployment, they will promote reducing the working hours, thus maintaining a stable employment rate, and allowing economic growth. This type of action benefits not only the consumer market but can also relief the pressure on the social safety net by reducing the unemployed [123]. More than 90 decades ago, Keynes made a famous prediction that the duration of the workweek would be of only 15h by the time his grandchildren came to age but more modern predictions consider that a reduction limited to 20–40% of the workweek would be more realistic [55, 123].

In many countries, non-standard employment workers will have minimum rights granted through government regulation after a series of disputes with their employers. This will improve the quality of services provided and reduce the insecurity of workers in this category. Disputes surrounding the employment contract of platform workers are already a reality around the world with mixed results and different proposals are already being put forward and can be expected to increase in the future to create at least some intermediary set of rights that place NSE workers in a situation of less insecurity than the current one [67].

By progressively increasing taxes for larger corporations, and taxing great fortunes and heritages, the government will distribute wealth to the poorer sections of society, including the promotion of basic income efforts. Wealth distribution efforts would allow the reduction of poverty and the stabilization of inequality. The implementation of international tax cooperation could also help with the reduction of inequality between developed and developing countries with the payment of universal basic income as a way of transferring resources from the first group to the second [124]. This is certainly a challenge that can be seen as a stretch considering the current reality of global cooperation, but even if no international circulation of financial resources is not possible in the future, at least some cooperation in terms of knowledge regarding actions to deal with the negative consequences of technological change can be considered in the future [124].

## Conclusion

Work has changed in the past, and it is continuously changing. Future studies are important to highlight which important changes must be made now to prevent future problems. Therefore, the future of work is a relevant subject for future study because we are currently going through the 4th Industrial Revolution—focused on robotics, AI, biotechnology, and nanotechnology—and a global pandemic which are events that produce fast and profound changes to society and work.

In this study, we analyzed the future of work on the horizon of 2050. We divided our scope into five groups: computerization/automation, employment, education, social welfare, and economy. As a result, we point out trend scenarios about the future of work—with some conflicting trends. Therefore, we developed and integrated these trends into three scenarios of Work in 2050: an optimistic/positive scenario, a pessimist/negative scenario, and a likely scenario which were presented in detail. The likely scenario combined the trends which we consider the most probable future outcomes, including the impacts caused by the COVID-19 pandemic.

Results show that computerization and automation continues to advance in industry and will reduce the demand for low-skill and low-wage jobs; non-standard employment tends to be better regulated, with minimum worker rights granted; new technologies will allow a transition to a personalized education process; the workload to the workers will reduce due this personalized education and computerization; automation will impact all types of work, and workers will receive knowledge-intensive training to make them able to perform the fewer available jobs; the self-employment and entrepreneurship will grow in the global labor market; society will demand more transparency and participation in political matters using new technologies; population will age and legislations will be amended so that pensions have increased ages; universal basic income would not reach its full potential, but income transfer programs will be implemented for the most vulnerable population; knowledge-intensive work and services will become more advanced; and extreme poverty will decrease but inequality will be slightly higher than it is nowadays.

This study contributes to the understanding of the current situation of work and its current future trends. We contribute to the discussion of problems related to work, to help decision-makers to understand them and take efficient actions to mitigate them.

## Data Availability

The datasets used and/or analyzed during the current study are available from the corresponding author on reasonable request.

## Abbreviations

AI: Artificial intelligence
AR: Augmented reality
GDP: Gross Domestic Product
HFI: Household Food Insecurity
IDH: Human Development Index
ICU: Intensive care unit
ILO: International Labor Organization
IoMT: Internet of Medical Things
IoT: Internet of Things
MOOC: Massive Open Online Courses
STEM: Science, Technology, Engineering, and Mathematics
TCC: Tele-critical care
UBA: Universal basic assets
UBI: Universal basic income
VR: Virtual reality
